# A Random Forest Based Risk Model for Reliable and Accurate Prediction of Receipt of Transfusion in Patients Undergoing Percutaneous Coronary Intervention

**DOI:** 10.1371/journal.pone.0096385

**Published:** 2014-05-09

**Authors:** Hitinder S. Gurm, Judith Kooiman, Thomas LaLonde, Cindy Grines, David Share, Milan Seth

**Affiliations:** 1 Department of Internal Medicine, Division of Cardiovascular Medicine, University of Michigan, Ann Arbor, Michigan, United States of America; 2 Department of Thrombosis and Hemostasis and Department of Nephrology, Leiden University Medical Center, Leiden, The Netherlands; 3 Department of Internal Medicine, St John Providence Health System, Detroit, Michigan, United States of America; 4 Department of Internal Medicine, Detroit Medical Center, Detroit, Michigan, United States of America; 5 Blue Cross Blue Shield of Michigan, Detroit, Michigan, United States of America; University of Louisville, United States of America

## Abstract

**Background:**

Transfusion is a common complication of Percutaneous Coronary Intervention (PCI) and is associated with adverse short and long term outcomes. There is no risk model for identifying patients most likely to receive transfusion after PCI. The objective of our study was to develop and validate a tool for predicting receipt of blood transfusion in patients undergoing contemporary PCI.

**Methods:**

Random forest models were developed utilizing 45 pre-procedural clinical and laboratory variables to estimate the receipt of transfusion in patients undergoing PCI. The most influential variables were selected for inclusion in an abbreviated model. Model performance estimating transfusion was evaluated in an independent validation dataset using area under the ROC curve (AUC), with net reclassification improvement (NRI) used to compare full and reduced model prediction after grouping in low, intermediate, and high risk categories. The impact of procedural anticoagulation on observed versus predicted transfusion rates were assessed for the different risk categories.

**Results:**

Our study cohort was comprised of 103,294 PCI procedures performed at 46 hospitals between July 2009 through December 2012 in Michigan of which 72,328 (70%) were randomly selected for training the models, and 30,966 (30%) for validation. The models demonstrated excellent calibration and discrimination (AUC: full model  = 0.888 (95% CI 0.877–0.899), reduced model AUC = 0.880 (95% CI, 0.868–0.892), p for difference 0.003, NRI = 2.77%, p = 0.007). Procedural anticoagulation and radial access significantly influenced transfusion rates in the intermediate and high risk patients but no clinically relevant impact was noted in low risk patients, who made up 70% of the total cohort.

**Conclusions:**

The risk of transfusion among patients undergoing PCI can be reliably calculated using a novel easy to use computational tool (https://bmc2.org/calculators/transfusion). This risk prediction algorithm may prove useful for both bed side clinical decision making and risk adjustment for assessment of quality.

## Introduction

Bleeding and transfusion after PCI have been associated with increased morbidity, short term and long term mortality and increased health care cost[1], [2], [3], [4]. Although there is considerable debate on the causal versus casual nature of the relation between bleeding and mortality, there is general consensus that bleeding is a negative outcome following PCI and is best avoided[5]. Assessment of transfusion after PCI as a quality measure is more complex since blood transfusion is clearly necessary in some patients, and occasionally may even be life-saving, whereas it may be avoidable in others[2]. Transfusion has been associated with several adverse outcomes and is associated with worsened short and long term survival in patients with acute coronary syndrome and following coronary revascularization[6], [7], [8]. There is increasing evidence that restrictive blood transfusion policies may be beneficial in patients with cardiac disease and there is increasing focus on transfusion as a quality improvement objective.

Considerable variation in transfusion rates have been identified across institutions and it remains unclear if this is driven by variations in case mix and/or practice[9]. Lack of a validated model to predict likelihood of transfusion serves as an impediment to benchmarking and guiding quality improvement. Further, such a model, if available could help guide individualized care and guide therapeutic strategies to reduce transfusion in patients who are most at risk.

The widespread use of computers in medical care has opened up the possibility of bed side application of more complex tools that leverage developments in statistical science, and facilitate use of algorithms that cannot be easily converted into risk scores[10], [11]. We have recently reported on such a tool for prediction of contrast induced nephropathy in patients undergoing PCI[12].

The goal of our work was to use a similar approach to develop a highly accurate model for prediction of transfusion using pre-procedural variables that are routinely collected in patients undergoing PCI, while retaining the advantages of bed side applicability. Further, we evaluated the impact of bleeding avoidance strategies on observed transfusion rates based on predicted transfusion risk.

## Methods

We developed and validated the transfusion model using data from the Blue Cross Blue Shield of Michigan cardiovascular consortium (BMC2), a quality improvement collaborative that tracks the inpatient outcome of consecutive patients undergoing PCI at all non-federal hospitals in the State of Michigan. The details of the BMC2 and its data collection and auditing process have been described previously[13], [14]. BMC2 registry is a clinical registry that tracks the outcome of all consecutive patients undergoing PCI at the participating institutions. Procedural data are collected using standardized data collection forms. Baseline data include clinical, demographic, procedural, and angiographic characteristics as well as medications used before, during, and after the procedure, and in-hospital outcomes. All data elements have been prospectively defined. In addition to a random audit of 2% of all cases, medical records of all patients undergoing multiple procedures or coronary artery bypass grafting (CABG) and of patients who died in the hospital are reviewed routinely to ensure data accuracy. The audit has revealed a data accuracy of over 95% for the study population.

The BMC2 registry and waiver of patient consent has been either approved by or the need for approval waived by the IRB at each of the participating hospitals. The University of Michigan has waived the need for IRB approval on all analysis that are performed using BMC2 data. The need for consent has been waived since all data are anonymous and no patient identifiers are collected.

The study population for this analysis included all consecutive patients who underwent PCI between July 2009 through December 2012. Patients who underwent coronary artery bypass grafting during the same hospitalization were excluded from the analysis since a post –operative transfusion could not be distinguished from post PCI transfusion. The choice of vascular access, procedural anticoagulation and decision to transfuse was as per the operator preference guided by institutional policy and practice.

### Study endpoints

The primary endpoint for our study was blood transfusion. Transfusion was defined as transfusion of packed red cells or whole blood after the PCI procedure but prior to hospital discharge irrespective of the total number of units transfused. Baseline hemoglobin was collected within a month of the procedure. Among patients who had multiple assessments of hemoglobin, the value closest to the time of the procedure was considered as the baseline value.

### Model development

The model was developed using a random forest method as previously described.[12] The study cohort was divided randomly into training and validation datasets, with 70% of procedures assigned to training, and the remaining 30% utilized for validation. A random forest regression model was trained for predicting transfusion using 45 baseline clinical variables including pre-procedural medications, with missing predictors imputed to be the overall median for continuous values and mode for categorical variables. Details of random forest methods have been described elsewhere[15]. Briefly random forest is an ensemble classification method that determines a consensus prediction for each observation by averaging the results of many individual recursive partitioning tree models. Each of the individual trees are fitted to a randomly selected subset of the observations, and utilize a random subset of the available predictors at each node as candidates for splitting. Random forests have been shown to have good predictive value, and are generally robust to issues of over-fitting, and missing data, and are particularly suited for evaluating a large number of possible predictors and exploiting potential interactions between predictors and their relationship with the outcome[16]. The transfusion outcome was entered as a continuous variable coded as 1 in patients who were transfused, and 0 for those not meeting the criteria to facilitate regression rather than classification modeling, so that estimated means (leaf node probabilities of transfusion) assigned to a given observation were then aggregated in the ensemble. To facilitate the development of an easy to use bedside tool, a reduced model was also trained using only the fourteen most important predictors as assessed in the full model by the incremental decrease in node impurity (residual sum of squares) associated with splitting on the predictor averaged over all trees in the ensemble.

### Model validation

The full and reduced models were evaluated in terms of discrimination and calibration in the validation dataset through evaluation of the area under the ROC curve (AUC), and by graphical examination of observed versus predicted transfusion rates after grouping observations by predicted risk (<1%, 1–2%, 2–3%, 3–5%, 5–10%, 10–15%, 15–20%, 20–30%, 30–40%, and >40%). The net reclassification index was used to compare full and reduced model performance, after classifying the predicted risk as low, medium and high; p-values and confidence intervals were obtained through bootstrapping[12], [17], [18]. Random forest estimates for observations in the validation dataset were scaled so that the overall predicted transfusion rate for the validation sample matched the overall transfusion rate observed in the training dataset.

The potential application of the tool for guiding individualized decision making was assessed by comparing the predicted and observed transfusion rates in the low, medium, and high risk categories in patients treated with heparin alone, heparin and platelet glycoprotein IIbIIIa inhibitor (GPI) and bivalirudin. For patients in each risk category, the unadjusted number needed to treat (NNT) with bivalirudin compared to GPI in order to prevent one transfusion was estimated as the inverse of the absolute difference in observed transfusion rates. A similar calculation was made for use of radial versus femoral access.

All analyses were performed in R version 2.14.1 using freely distributed contributed packages[19], [20].

## Results

Our study cohort comprised of 103,294 (99%) of 104,408 procedures performed across Michigan between July 2009 through December 2012. We excluded 1047 (1%) patients since they underwent CABG during the same hospitalization (n = 1,018) or when post procedural CABG data were not available (n = 29), and 67 patients for whom post-procedural transfusion data were not available.

The training dataset consisted of 72,328 PCI procedures of which 2156 (3.0%) were accompanied by transfusion, and the validation dataset of 30,966 procedures of which 922 (3.0%) were followed by a transfusion. All baseline variables presented in Table 1 were included in the full random forest model. The training and validation datasets were similar in terms of baseline covariates (Table 2). The variables with the largest model determined importance are listed in Table 3, and Table 4 provides their distribution in training dataset patients both with and without transfusion. This set of predictors was used to fit the reduced random forest model that is available for use at https://bmc2.org/calculators/transfusion.

**Table 1:** Patient/procedural characteristics included in Full model.

History and risk factors:						Clinical presentation:
Current Smoker (w/in 1 year)						Cardiomyopathy/LV systolic dysfunction
Former smoker						Pre-operative evaluation prior to Non-Cardiac Surgery
Hypertension						Cardiogenic Shock w/in 24 hours prior to presentation
Dyslipidemia						Cardiac Arrest w/in 24 hours prior to presentation
Family history of Premature CAD						Stress/imaging study performed
Prior MI						Exercise stress test results
Prior Heart Failure						Stress Echo imaging results
Prior Valve Surgery/Procedure						Cardiac CTA performed
Prior PAD						Cardiac CTA results
Prior PCI						Coronary Calcium score
Prior CABG						CAD Presentation
Prior ICD						Anginal Classification w/in 2 weeks
Height						NYHA class w/in 2 weeks
Weight						
Age						**Pre-Procedural Lab values:**
Currently on Dialysis						Creatine kinase -MB
Cerebrovascular disease						Troponin I
Chronic lung disease						Troponin T
Diabetes/Diabetes Therapy (Diet, Oral Rx, Insulin)						Creatinine
Gastro-intestinal Bleeding						Hemoglobin
Valve disease						
Surgery within the prior 7 days						
Atrial Fibrillation						
Cardiac transplant						
Cardiac arrest						

**Table 2:** Characteristics of patients in the training and the validation cohort.

Characteristic	Training	Validation	p-value	Abs. Std. diff
Number of patients:	72,376	30,985	NA	NA
BMI	30.62±7.49	30.51±7.54	p = 0.040	1.40
Age	64.86±12.11	64.91±12.08	p = 0.551	0.40
Sex: Male	47,888/72,375 (66.2%)	20,329/30,984 (65.6%)	p = 0.084	1.17
Race – White	62,566/72,376 (86.4%)	26,782/30,985 (86.4%)	p = 0.964	0.03
Current/Recent Smoker (w/in 1 year)	21,172/72,321 (29.3%)	9,208/30,962 (29.7%)	p = 0.133	1.02
Hypertension	61,598/72,325 (85.2%)	26,380/30,968 (85.2%)	p = 0.946	0.05
Dyslipidemia	59,811/72,279 (82.8%)	25,777/30,953 (83.3%)	p = 0.039	1.41
Family History of Premature CAD	14,468/72,350 (20.0%)	6,227/30,973 (20.1%)	p = 0.693	0.27
Prior MI	25,336/72,355 (35.0%)	10,956/30,979 (35.4%)	p = 0.281	0.73
Prior Heart Failure	11,261/72,330 (15.6%)	4,851/30,966 (15.7%)	p = 0.695	0.27
Prior Valve Surgery/Procedure	1,132/72,327 (1.6%)	539/30,966 (1.7%)	p = 0.041	1.38
Prior PCI	32,606/72,366 (45.1%)	14,043/30,980 (45.3%)	p = 0.420	0.55
Prior CABG	13,650/72,355 (18.9%)	5,788/30,976 (18.7%)	p = 0.498	0.46
Height	171.05±10.59	170.95±10.59	p = 0.181	0.91
Weight	89.60±21.44	89.20±21.03	p = 0.005	1.89
Cerebrovascular Disease	11,031/72,325 (15.3%)	4,743/30,964 (15.3%)	p = 0.788	0.18
Peripheral Arterial Disease	11,848/72,325 (16.4%)	5,171/30,970 (16.7%)	p = 0.211	0.85
Chronic Lung Disease	13,358/72,328 (18.5%)	5,770/30,962 (18.6%)	p = 0.526	0.43
Diabetes Mellitus	27,229/72,362 (37.6%)	11,491/30,975 (37.1%)	p = 0.106	1.10
Diabetes Therapy: Insulin	11,301/27,132 (41.7%)	4,757/11,463 (41.5%)	p = 0.780	0.31
CAD Presentation: No symptom, no angina	5,199/72,351 (7.2%)	2,276/30,979 (7.3%)	p = 0.360	0.62
CAD Presentation: Stable angina	12,491/72,351 (17.3%)	5,405/30,979 (17.4%)	p = 0.477	0.48
CAD Presentation: Unstable angina	27,978/72,351 (38.7%)	11,905/30,979 (38.4%)	p = 0.467	0.49
CAD Presentation: Non-STEMI	14,093/72,351 (19.5%)	6,001/30,979 (19.4%)	p = 0.689	0.27
CAD Presentation: ST-Elevation MI (STEMI) or equivalent	10,875/72,351 (15.0%)	4,640/30,979 (15.0%)	p = 0.827	0.15
Heart Failure w/in 2 Weeks	7,422/72,340 (10.3%)	3,161/30,971 (10.2%)	p = 0.795	0.18
NYHA Class w/in 2 Weeks: Class I	546/7,361 (7.4%)	227/3,141 (7.2%)	p = 0.732	0.73
NYHA Class w/in 2 Weeks: Class II	1,793/7,361 (24.4%)	738/3,141 (23.5%)	p = 0.344	2.02
NYHA Class w/in 2 Weeks: Class III	2,860/7,361 (38.9%)	1,184/3,141 (37.7%)	p = 0.264	2.38
NYHA Class w/in 2 Weeks: Class IV	2,162/7,361 (29.4%)	992/3,141 (31.6%)	p = 0.024	4.81
Cardiomyopathy or Left Ventricular Systolic Dysfunction	7,630/72,359 (10.5%)	3,370/30,979 (10.9%)	p = 0.111	1.08
Pre-operative Evaluation Before Non-Cardiac Surgery	1,494/72,335 (2.1%)	686/30,968 (2.2%)	p = 0.125	1.04
Cardiogenic Shock w/in 24 Hours	1,241/72,350 (1.7%)	543/30,978 (1.8%)	p = 0.671	0.29
Cardiac Arrest w/in 24 Hours	1,326/72,325 (1.8%)	553/30,969 (1.8%)	p = 0.599	0.36
Fluoroscopy Time	14.72±11.28	14.74±11.69	p = 0.716	0.25
Fluoroscopy Dose	721.59±1277.24	719.50±1189.73	p = 0.941	0.17
Contrast Volume	191.58±78.34	190.62±77.84	p = 0.072	1.22
IABP	1,721/72,351 (2.4%)	761/30,975 (2.5%)	p = 0.452	0.51
Other Mechanical Ventricular Support	512/72,341 (0.7%)	217/30,968 (0.7%)	p = 0.901	0.08
Arterial Access Site: Femoral	64,854/72,342 (89.6%)	27,763/30,969 (89.6%)	p = 0.994	0.00
Arterial Access Site: Radial	7,195/72,342 (9.9%)	3,078/30,969 (9.9%)	p = 0.973	0.02
PCI Status: Elective	29,810/72,327 (41.2%)	12,886/30,948 (41.6%)	p = 0.207	0.86
PCI Status: Urgent	31,116/72,327 (43.0%)	13,251/30,948 (42.8%)	p = 0.543	0.41
PCI Status: Emergency	11,270/72,327 (15.6%)	4,753/30,948 (15.4%)	p = 0.362	0.62
PCI Status: Salvage	131/72,327 (0.2%)	58/30,948 (0.2%)	p = 0.829	0.15
Pre-PCI Left Ventricular Ejection Fraction	51.95±12.69	52.08±12.67	p = 0.186	1.02
CK-MB Pre-Procedure	27.41±66.99	26.14±60.42	p = 0.202	1.99
CK Pre-Procedure Drawn and Normal	6,574/19,792 (33.2%)	2,877/8,551 (33.6%)	p = 0.481	0.91
Troponin I Pre-Procedure	4.49±21.54	4.13±15.73	p = 0.053	1.94
Troponin T Pre-Procedure	0.85±6.54	0.71±3.26	p = 0.190	2.61
Pre-Procedure Creatinine	1.16±0.94	1.16±0.97	p = 0.529	0.43
Pre-Procedure Hemoglobin	13.42±1.89	13.43±1.91	p = 0.282	0.74
CK-MB Post-Procedure	57.77±131.53	56.48±119.93	p = 0.406	1.02
Troponin I Post-Procedure	27.88±62.48	28.24±68.46	p = 0.694	0.56
Troponin T Post-Procedure	6.24±26.14	5.50±15.83	p = 0.272	3.45
Post-Procedure Creatinine	1.19±1.08	1.20±1.10	p = 0.539	0.44
Post-Procedure Hemoglobin	12.24±1.92	12.24±1.94	p = 0.944	0.05
Myocardial Infarction (Biomarker Positive)	1,432/72,318 (2.0%)	660/30,969 (2.1%)	p = 0.114	1.06
RBC/Whole Blood Transfusion	2,156/72,328 (3.0%)	922/30,966 (3.0%)	p = 0.977	0.02
Hemoglobin Prior to Transfusion	8.10±1.25	8.08±1.16	p = 0.657	1.73
Bleeding Event w/in 72 Hours	1,796/72,327 (2.5%)	767/30,967 (2.5%)	p = 0.952	0.04
Discharge Status: Alive	71,352/72,376 (98.6%)	30,554/30,985 (98.6%)	p = 0.766	0.20

**Table 3:** Patient/procedural characteristics selected for reduced model.

Reduced Model variables:	
**History and risk factors**	**Clinical presentation**
Height	Heart Failure w/in 2 Weeks
Weight	CAD Presentation
Age	Anginal Classification
History of Chronic Lung Disease	Cardiogenic Shock (within 24 hours prior to or at start of PCI)
Diabetes/Diabetes Therapy (Diet, Oral therapy, Insulin)	
	
**Pre-Procedural Lab values**	
Creatine kinase-MB	
Troponin I	
Troponin T	
Creatinine	

**Table 4:** Distribution of abbreviated model covariates by transfusion status in the training dataset.

Characteristic	No Transfusion	Transfusion	p-value	Abs. Std. diff
				
Age	64.72±12.02	71.20±12.46	p<0.001	52.9
Height	171.11±10.53	165.94±11.26	p<0.001	47.4
Weight	89.44±20.94	81.34±22.59	p<0.001	37.2
Chronic Lung Disease	5,496/30,023 (18.3%)	267/920 (29.0%)	p<0.001	25.4
Diabetes Mellitus	11,032/30,034 (36.7%)	450/922 (48.8%)	p<0.001	24.6
Diabetes Therapy: None	465/11,007 (4.2%)	19/447 (4.3%)	p = 0.979	0.1
Diabetes Therapy: Diet	661/11,007 (6.0%)	24/447 (5.4%)	p = 0.578	2.7
Diabetes Therapy: Oral	5,337/11,007 (48.5%)	157/447 (35.1%)	p<0.001	27.3
Diabetes Therapy: Insulin	4,508/11,007 (41.0%)	246/447 (55.0%)	p<0.001	28.5
Diabetes Therapy: Other	36/11,007 (0.3%)	1/447 (0.2%)	p = 0.706	2.0
Cardiogenic Shock w/in 24 hours or at start of PCI	607/30,005 (2.0%)	149/921 (16.2%)	p<0.001	50.8
CAD Presentation: No symptom, no angina	2,216/30,038 (7.4%)	58/922 (6.3%)	p = 0.213	4.3
CAD Presentation: Symptom unlikely to be ischemic	736/30,038 (2.5%)	14/922 (1.5%)	p = 0.070	6.7
CAD Presentation: Stable angina	5,339/30,038 (17.8%)	64/922 (6.9%)	p<0.001	33.4
CAD Presentation: Unstable angina	11,661/30,038 (38.8%)	238/922 (25.8%)	p<0.001	28.1
CAD Presentation: Non-STEMI	5,719/30,038 (19.0%)	276/922 (29.9%)	p<0.001	25.5
CAD Presentation: ST-Elevation MI (STEMI) or equivalent	4,367/30,038 (14.5%)	272/922 (29.5%)	p<0.001	36.7
Anginal Classification w/in 2 Weeks: No symptoms	3,370/29,993 (11.2%)	137/919 (14.9%)	p<0.001	10.9
Anginal Classification w/in 2 Weeks: CCS I	1,043/29,993 (3.5%)	15/919 (1.6%)	p = 0.002	11.7
Anginal Classification w/in 2 Weeks: CCS II	4,711/29,993 (15.7%)	71/919 (7.7%)	p<0.001	25.0
Anginal Classification w/in 2 Weeks: CCS III	10,451/29,993 (34.8%)	229/919 (24.9%)	p<0.001	21.8
Anginal Classification w/in 2 Weeks: CCS IV	10,418/29,993 (34.7%)	467/919 (50.8%)	p<0.001	32.9
Heart Failure w/in 2 Weeks	2,840/30,031 (9.5%)	316/921 (34.3%)	p<0.001	63.0
CK-MB Pre-Procedure	25.99±60.80	30.58±50.70	p = 0.201	8.2
Troponin I Pre-Procedure	3.97±15.56	7.58±18.90	p<0.001	20.8
Troponin T Pre-Procedure	0.66±3.18	1.95±4.73	p = 0.006	32.0
Pre-Procedure Creatinine	1.14±0.93	1.75±1.70	p<0.001	44.0
Pre-Procedure Hemoglobin	13.51±1.84	10.85±2.10	p<0.001	134.8

**Figure 1:**
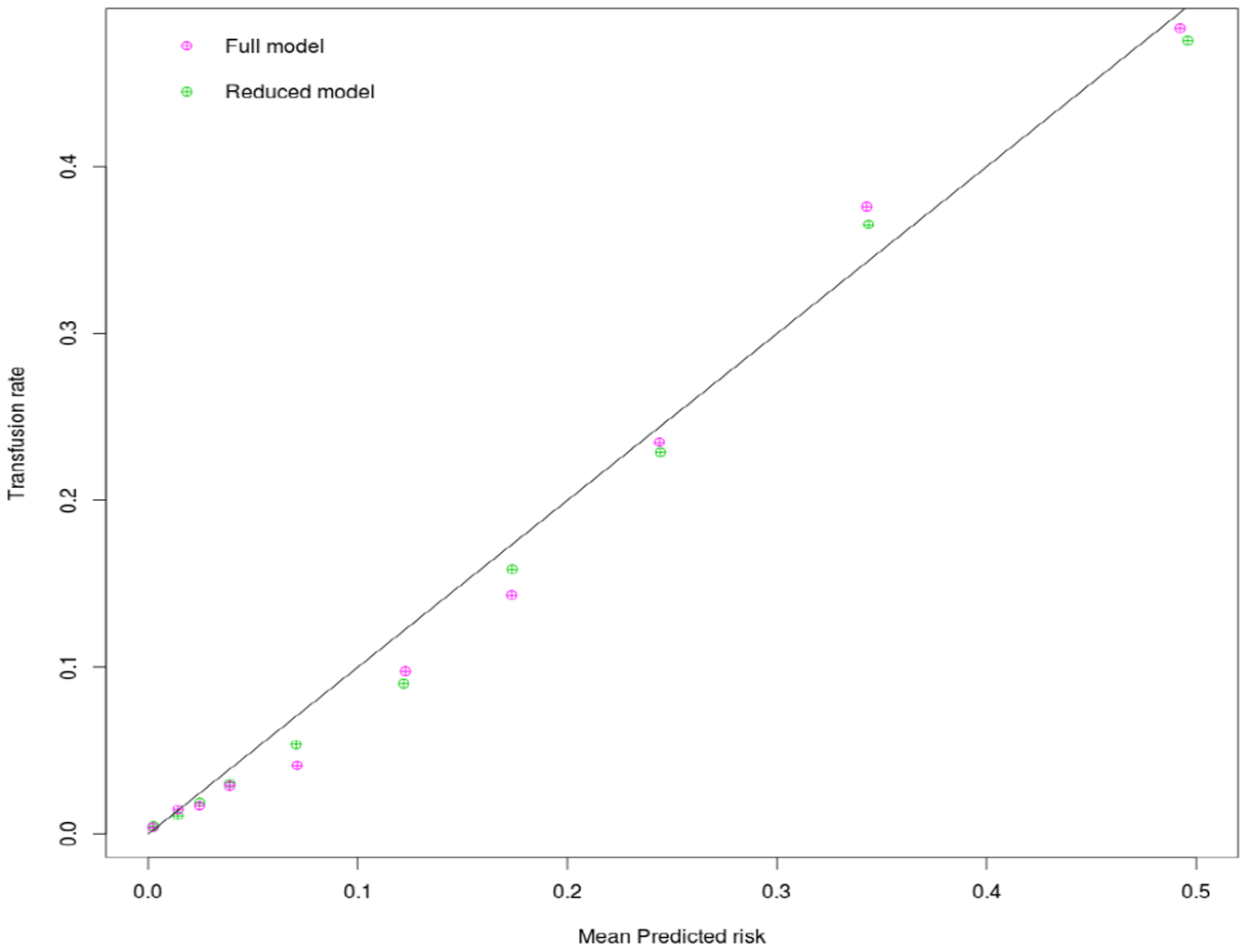
Calibration plot depicting observed transfusion across predicted risk using the full and the abbreviated model.

When evaluated in the validation dataset, both models provide good discrimination for transfusion, with the full model having a small but statistically significant advantage in AUC (full model AUC: 0.888 [95% CI, 0.877–0.899], reduced model AUC: 0.880 [95% CI, 0.868–0.892]. p for difference  = 0.003). Both models demonstrated high calibration (Figure 1) with good concordance between observed and predicted transfusion rates.

The full and reduced model predictions were grouped into low risk (<1%), intermediate risk (1–5%), and high risk (>5%) categories and the number of patients along with the observed transfusion rate in each group is presented in Table 5. The patients in the highest risk category comprised one sixth of the total population but received over 75% of the transfusions. The net reclassification improvement statistic for the full model relative to the reduced model for these categories was small but statistically significant (NRI: 2.77%, [0.62–5.06%], p = .007).

**Table 5:** Model Comparison by risk category.

**Reduced Model**					
**Estimated risk:**	**# of Procedures:**	**# of Transfusions:**	**Transfusion rate:**	**% of procedures:**	**% of Transfusions:**
Low (<1%)	18,239	89	0.49%	58.90%	9.65%
Medium (1–5%)	7,648	141	1.84%	24.70%	15.29%
High (>5%)	5,079	692	13.62%	16.40%	75.05%
**Full Model**					
**Estimated risk:**	**# of Procedures:**	**# of Transfusions:**	**Transfusion rate:**	**% of procedures:**	**% of Transfusions:**
Low (<1%)	18,479	76	0.41%	59.68%	8.24%
Medium (1–5%)	7,144	138	1.93%	23.07%	14.97%
High (>5%)	5,343	708	13.25%	17.25%	76.79%

**Figure 2:**
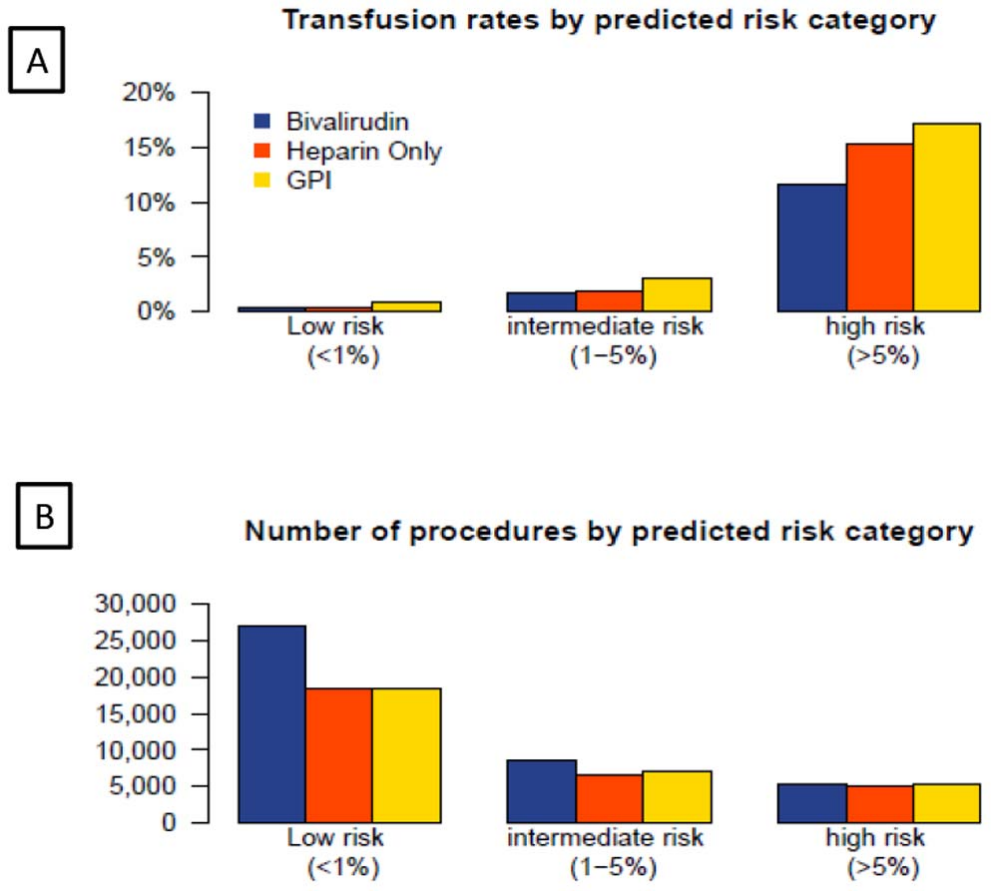
The observed transfusion rates across the three predicted risk categories in patients treated with heparin only, bivalirudin and platelet glycoprotein IIbIIIa inhibitor (with heparin) is depicted in panel A. Panel B depicts the total number of patients treated with each anticoagulation strategy across the three transfusion risk groups.

**Figure 3:**
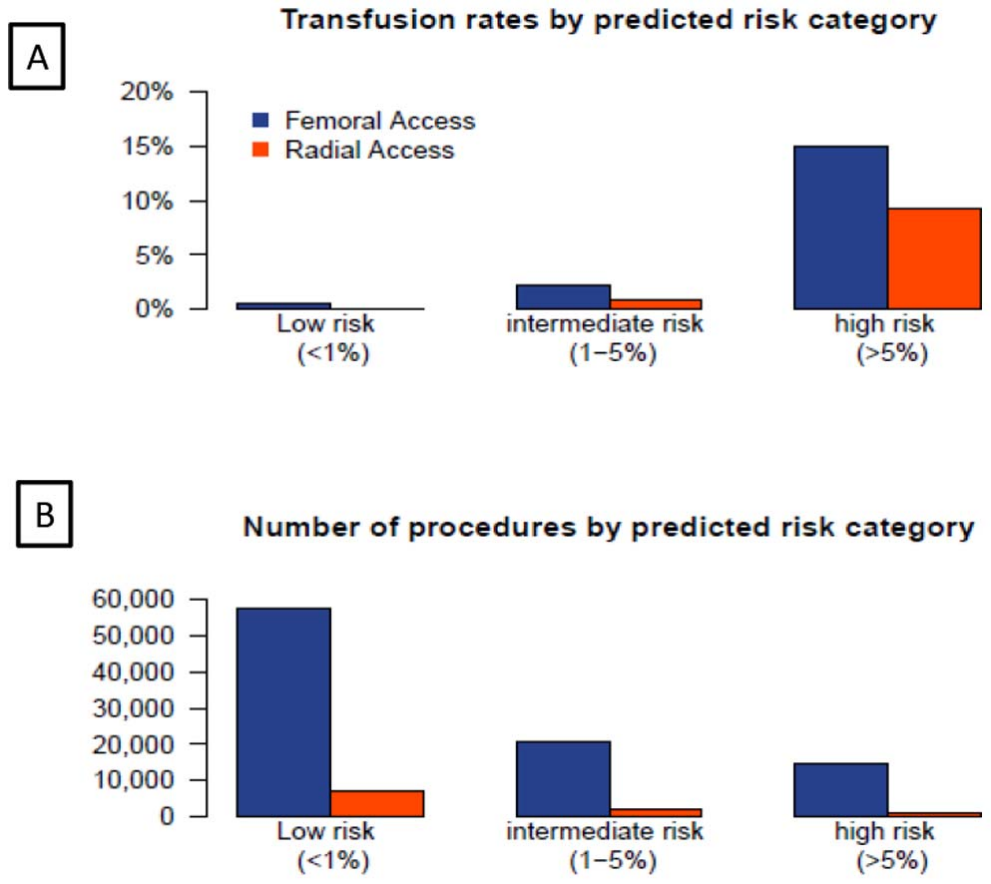
The observed transfusion rates across the three predicted risk categories in patients treated with femoral versus radial access is depicted in panel A. Panel B depicts the total number of patients treated with the two access routes across the three transfusion risk groups. There is an inverse association between predicted transfusion risk and access route with radial access being more commonly used in the low risk patients.

Figure 2 depicts the observed transfusion rates across the three predicted risk categories in patients treated with heparin only, bivalirudin and GPI (with heparin).The use of GPI is associated with the highest transfusion rates while bivalirudin was associated with the lowest transfusion rates overall, although in the lowest risk category the transfusion rates for all three anticoagulant strategies were very small, so that the absolute differences were not clinically meaningful (<.5%). The highest risk group by contrast, demonstrated the greatest absolute difference in bleeding (>5%) so that only 19 patients would need to be treated with bivalirudin instead of GPI to prevent one transfusion (Table 6). Figure 3 provides transfusion rates by risk categories for patients with radial and femoral vascular access. When the impact of access site on transfusion was considered, the greatest benefit of radial access was seen in patients in the highest risk category although a lower transfusion rate was observed with radial access in all risk groups. The number needed to treat with radial versus femoral approach to prevent one transfusion was 18 for the highest risk category and 244 for the lowest risk cohort.

**Table 6:** Projected numbers needed to treat (NNT) to prevent one transfusion across categories of predicted risk.

Predicted risk	<1%	1–5%	>5%
NNT with Bivalirudin instead of Heparin only to prevent one transfusion	1721	664	28
NNT with Bivalirudin instead of Glycoprotein IIbIIIa inhibitor and Heparin to prevent one transfusion	202	76	19
NNT with radial access versus femoral approach to prevent one transfusion	244	69	18

## Discussion

The key finding of our study is that the risk of transfusion in patients undergoing PCI can be reliably estimated using standard clinical and laboratory variables that are routinely collected in this population. Secondly, this tool helps identify patient subgroups that are at higher or lower risk of needing a transfusion and can therefore guide appropriate choice of pharmacotherapy or vascular access in a cost effective fashion. The robust discrimination and calibration of this method, combined with the ease of use for simplified bedside prediction, makes this model an easy tool for routine clinical practice.

Risk stratification models have been advocated for two broad usages: patient level decision making (for guiding informed consent, and therapeutic decision-making) and risk adjustment for assessment of quality of care. Our model has several advantages that make it especially suited for these purposes.

First, to the best of our knowledge, this is the only model to predict transfusion that has been developed and validated on a contemporary patient population. Secondly, the model has a very high discrimination that should improve reliability and accuracy of risk estimates. While there are no contemporary models to predict transfusion, the NCDR bleeding prediction model is perhaps the closest in its clinical application[21]. The modest discrimination of that model (C statistics of 0.72) raises concerns about misclassification when it is applied for individualized decision making. Thirdly, our model should be generalizable to routine clinical practice since it is developed and validated on all consecutive patients treated in Michigan and reflects contemporary practice across multiple institutions and operators. The model is based only on pre-procedure variables, and thus can be used for risk stratification prior to the procedure. This can help facilitate better informed consent as well as consideration of alternate therapeutic strategies that would minimize the risk of bleeding and the resultant need for transfusion.

Unlike traditional risk scores, our model requires a computer for calculation and cannot be converted into a bedside arithmetic risk score. While, the need to favor simplicity over accuracy might have been reasonable in the past, these considerations should no longer be relevant in the era of the widespread use of smart devices and electronic medical records. We developed two different models, with the full model providing a slightly greater discrimination compared with the abbreviated model. In the ideal world, models like ours would be embedded in the electronic medical record, and would be an integral part of the clinical workflow, providing physicians and patients with accurate risk estimates. All the predictors in this model are routinely ascertained and are embedded in the templates that are used for the documentation of the initial history and physical assessment of a patient being evaluated for PCI. Therefore, real time automatic risk estimation is feasible and hopefully will be adapted in the near future by the vendors of electronic medical record systems.

We envision multiple application of this model. The BMC2 consortium is using this model for calculating risk adjusted transfusion rates for physicians and operators and this will guide quality improvement efforts. Initial application of this approach has identified institutions where the rate of transfusion is significantly greater than expected and these hospitals have initiated focused efforts geared towards reducing transfusion. Secondly, the model can be used to personalize the consent process and the patient provided with their personalized risk estimate rather than the standard average risk of bleeding. Thirdly, the model helps identify the 16% of patients who are most likely to need transfusion and thus the ideal subset for use of strategies that have been proven to reduce the risk of transfusion such as bivalirudin or use of radial approach. Conversely, the model helps identify the large subset of patients who are at the extremely low risk and in whom the use of such therapies may not be that beneficial or cost effective. The use of the model to target therapies like bivalirudin (with demonstrated reduction in transfusion but increased expense relative to heparin) to the highest risk patients, while avoiding it in the low risk patients has the potential to reduce both cost and complications and should be evaluated in future studies. Use of the NCDR bleeding model in this fashion has been recently demonstrated to be associated with clinically meaningful reductions in bleeding and transfusion and it likely that the use of our model with its greater accuracy would enhance those benefits[22]. In our exploratory analysis, we demonstrate that the absolute benefit of bleeding avoidance therapies is dependent on both the baseline bleeding risk as well as the type of therapy used. As expected, radial access is the most effective approach towards preventing transfusion with a number needed to treat of 19 among patients at high risk while the benefit is less impressive and of uncertain clinical significance in patients at low risk of bleeding. This is also evident in the comparison of heparin and bivalirudin in low risk patients where the absolute difference in events is too small to be clinically meaningful and many institutions may not be able to prevent one transfusion in a year even if they treated all their low risk patients with bivalirudin instead of heparin. It is expected that as clinicians use this tool in practice, other uses will emerge that will lead to further optimization of patient care, as well as modification and refinement of the prediction tool.

Like most observational studies, our study findings must be evaluated with certain caveats. We developed a model to predict transfusion, which is distinct from bleeding. While both bleeding and transfusion can be considered negative outcomes following PCI, transfusion, unlike bleeding, is occasionally necessary and cannot be considered a never event. Secondly, while bleeding that does not require transfusion is associated with adverse long term outcomes, it is unclear if the relationship is causal. On the other hand transfusion, if not needed, is best avoided both due to its negative health impact, and the associated cost. A model for predicting transfusion thus can help guide quality improvement as well as guide clinical practice. Furthermore, the decision to transfuse in our population was clinically driven and may vary from physician to physician and across institutions. However, we believe this makes our model more generalizable to routine clinical care since it reflects findings from contemporary practice across the entire patient population undergoing PCI in Michigan.

## Conclusion

We have developed a simple tool for accurately predicting risk of transfusion among patients undergoing PCI. This risk prediction algorithm may prove useful for both bed side clinical decision making and risk adjustment for assessment of quality.
